# Acetylation reduces SOX9 nuclear entry *and ACAN* gene transactivation in human chondrocytes

**DOI:** 10.1111/acel.12456

**Published:** 2016-02-22

**Authors:** Michal Bar Oz, Ashok Kumar, Jinan Elayyan, Eli Reich, Milana Binyamin, Leonid Kandel, Meir Liebergall, Juergen Steinmeyer, Veronique Lefebvre, Mona Dvir‐Ginzberg

**Affiliations:** ^1^ Laboratory of Cartilage BiologyInstitute of Dental SciencesHebrew University of JerusalemJerusalemIsrael; ^2^ Joint Replacement and Reconstructive Surgery UnitOrthopaedic Surgery ComplexHadassah Mount Scopus HospitalJerusalemIsrael; ^3^ Laboratory for Experimental OrthopaedicsDepartment of Orthopaedic SurgeryUniversity Hospital Giessen & Marburg GmbHGießenGermany; ^4^ Cleveland Clinic Lerner Research InstituteClevelandOHUSA

**Keywords:** Aging, osteoarthritis, cartilage, aggrecan, nucleus, SOX9, SIRT1, acetylation

## Abstract

Changes in the content of aggrecan, an essential proteoglycan of articular cartilage, have been implicated in the pathophysiology of osteoarthritis (OA), a prevalent age‐related, degenerative joint disease. Here, we examined the effect of SOX9 acetylation on *ACAN* transactivation in the context of osteoarthritis. Primary chondrocytes freshly isolated from degenerated OA cartilage displayed lower levels of *ACAN* mRNA and higher levels of acetylated SOX9 compared with cells from intact regions of OA cartilage. Degenerated OA cartilage presented chondrocyte clusters bearing diffused immunostaining for SOX9 compared with intact cartilage regions. Primary human chondrocytes freshly isolated from OA knee joints were cultured in monolayer or in three‐dimensional alginate microbeads (3D). SOX9 was hypo‐acetylated in 3D cultures and displayed enhanced binding to a −10 kb *ACAN* enhancer, a result consistent with higher *ACAN* mRNA levels than in monolayer cultures. It also co‐immunoprecipitated with SIRT1, a major deacetylase responsible for SOX9 deacetylation. Finally, immunofluorescence assays revealed increased nuclear localization of SOX9 in primary chondrocytes treated with the NAD SIRT1 cofactor, than in cells treated with a SIRT1 inhibitor. Inhibition of importin β by importazole maintained SOX9 in the cytoplasm, even in the presence of NAD. Based on these data, we conclude that deacetylation promotes SOX9 nuclear translocation and hence its ability to activate *ACAN*.

## Introduction

Articular cartilage ensures smooth movement of bones facing each other in joints. This essential tissue is built during development and maintained throughout adulthood by its resident cells, chondrocytes, whose lineage identity and differentiation are chiefly governed by the transcription factor SOX9 (Akiyama *et al*., 2002; Akiyama & Lefebvre, 2011) *SOX9* deficiency has been linked postnatally to idiopathic chondrodysplasia (dwarfism), intervertebral disk and articular cartilage degeneration diseases, namely osteoarthritis (OA) (Henry *et al*., 2012) While developmental defects directly or indirectly due to improper function or expression of *SOX9* are rare, it is believed that improper function or expression of *SOX9* might directly or indirectly contribute to the pathogenetic mechanisms that underlie cartilage degeneration in OA, a disease that affects the majority of the elderly and considerably reduces life's quality (Gabriel *et al*., 1997) This disease remains largely incurable today, and this is mainly due to insufficient understanding of the mechanisms underlying cartilage formation, adult maintenance and osteoarthritic degeneration.

Previous studies uncovered a key role for the nicotinamide adenine dinucleotide (NAD)‐dependent class III protein deacetylase SIRTUIN 1 (SIRT1) in cartilage homeostasis and gene expression (Dvir‐Ginzberg *et al*., 2008; Oppenheimer *et al*., 2014) While SIRT1 was shown to induce transcriptional repression in many cell types (Carafa *et al*., 2012), the unexpected, contrasting discovery was made that it strikingly potentiated SOX9 gene targets (i.e. *ACAN* and *COL2A1*) in human chondrocytes (Dvir‐Ginzberg *et al*., 2008; Dvir‐Ginzberg & Steinmeyer, 2013; Oppenheimer *et al*., 2014). *In vitro* studies showed that SIRT1 bound to and deacetylated SOX9, but did not affect SOX9 binding to the *COL2A1* enhancer, as observed by chromatin immunoprecipitation assays of chondrocytes overexpressing wild‐type SIRT1 and a dominant negative H355Y SIRT1 mutant (Dvir‐Ginzberg *et al*., 2008). Further studies suggested that most of SIRT1 action on *COL2A1* expression occurred through recruitment of the histone methyl‐transferase SET7/9 and other histone acetyl transferases (i.e. P300 and GCN5) to the promoter and enhancer sites of the gene (Dvir‐Ginzberg *et al*., 2008; Oppenheimer *et al*., 2014). On the other hand, the mechanisms whereby SIRT1 may regulate ACAN expression are yet unknown.

More recently, it was shown that the incubation of mesenchymal stem cells with resveratrol, a SIRT1 activator, resulted in enhanced levels of cartilage‐specific proteoglycans (CSPGs), collagen type II and SOX9 (Buhrmann *et al*., 2014). This study also revealed binding of SIRT1 to SOX9 in mesenchymal stem cells undergoing chondrogenic differentiation as well as in primary chondrocytes (Buhrmann *et al*., 2014), a result consistent with our previous observations (Dvir‐Ginzberg *et al*., 2008)*. In vivo* studies showed that loss of SIRT1 enzymatic function resulted in reduced levels of collagen type II and aggrecan in articular cartilage (Gabay *et al*., 2013). Moreover, examination of mice undergoing aging showed a correlation between SIRT1 cleavage and inactivation, reduced levels of collagen type II and aggrecan, and enhanced OA severity (Gabay *et al*., 2012). Overall, emerging *in vitro* and *in vivo* evidence supports the concept that loss of SIRT1 or its inactivation contributes to OA severity in an age‐ or injury‐dependent manner (Gabay *et al*., 2012, 2013; Matsuzaki *et al*., 2014).

We here aimed to decipher the mechanisms by which SIRT1 might regulate *ACAN* expression via SOX9. To this end, we examined SOX9 acetylation level and binding to a previously described *ACAN* enhancer,(Han & Lefebvre, 2008), in various experimental settings, including OA articular cartilage. To further understand the role of SOX9 acetylation, we aimed to investigate the impact of this protein modification on the stability and nuclear trafficking of SOX9.

## Materials and methods

### Mice experiments

Experimental procedures involving mice (CD1/129J) were carried out in accordance with NIH Committees for animal use and care (ARAC guidelines) and based on AAALAC (Association for Assessment and Accreditation of Laboratory Animal Care International) guidelines. Mice were subjected to 12‐h light/dark cycles and received food and water *ad libitum*. Following mating, females were sacrificed progeny at embryonic day 17 which were harvested for analysis.

### Cell cultures and transfection procedures

Primary chondrocyte cultures were obtained from OA donors in accordance with Hadassah Medical Center Institutional Review Board approval and in accordance with the Helsinki Declaration of ethical principles for medical research involving human subjects. End‐stage OA patients were diagnosed based on the severity of pain and restricted daily function, confirmed by degree 3–4 KL scores on standing anteroposterior X‐rays. A formal written informed consent was obtained from osteoarthritic (OA) donors undergoing total knee arthroplasty (*n* = 40 end‐stage osteoarthritic donors; mean age: 72 years; mean body mass index: 31.5 kg m^−2^; 60% female donors). Age‐matched non‐OA cartilage was obtained from National Disease Research Interchange (NDRI, Philadelphia, PA, USA).

Human chondrocytes were isolated and plated as described by Dvir‐Ginzberg *et al*. (2008). Cells were plated in 14‐cm^2^ tissue culture dishes at a concentration of 1.5 × 10^6^ cells per dish and were grown to confluence (passage 0 or 1) in DMEM (Sigma‐Aldrich, St Louis, MI, USA) containing 10% FCS and 1% penicillin–streptomycin (Beit‐Haemek Kibbutz, Israel). Cells were cultured in standard incubation conditions (37 °C, 5% CO_2_) until confluence.

OA‐derived cartilage possessing visible lesions and fissure were categorized as degenerative cartilage, while smooth hyaline surfaces were characterized as intact cartilage. Equal weight (1–3 g) of intact or degenerated cartilage tissues were dissected and processed for histology or subject to fresh chondrocytes isolation for downstream qRT–PCR or immunoblot analysis. Human embryonic kidney cell lines (HEK293) were obtained from ATCC and transfected with PolyJet transfection reagents based on the manufacturer's guidelines (SignaGen Laboratories, Rockville, MD, USA).

### Encapsulation of chondrocytes in alginate beads

Alginate microbead encapsulation was performed as described by Oppenheimer *et al*. (2014). Briefly, first‐passage (P1) cultured human chondrocytes were mixed with a 1.25% sodium alginate solution to obtain a final concentration of 1 × 10^6^ cells mL^−1^. The cell suspension was added dropwise using a 23‐gauge needle into a 102 mm CaCl_2_ solution and was set to polymerize with constant stirring for 10 min at RT. Microbeads were subsequently washed with 0.9% NaCl during two consecutive 5‐min agitations. The three‐dimensional (3D) microbead and monolayer (2D) cultures were maintained for 2 weeks in standard conditions.

For mechanical loading experiments, 12‐well plates containing 20 alginate beads with a total of 4 million cells per well were used. During the culture period of 2 days, multiwell plates with alginate encapsulated chondrocytes were subjected five times to hydrostatic pressure induced by intermittently applied centrifugation steps lasting five or 30 min. Hydrostatic pressure of 0.05 or 0.1 MPa was applied by centrifugation of multiwell plates in an Eppendorf centrifuge (model 5810R, rotor model A‐4‐62) at 66g or 127 g, respectively. Mega pascal (MPa) units were used to measure the extent of hydrostatic pressure employed during centrifugation regimen. MPa was calculated based on the formula (1) in (Detzel & Van Wie, 2011). Encapsulated chondrocytes were released from alginate beads using a depolymerization solution (55 mm sodium citrate, 30 mm EDTA, 150 mm NaCl, 10 mm HEPES, pH = 7.2) and subsequent centrifugation (240g, 5 min) to obtain cell pellets. Protein, mRNA and chromatin were isolated from alginate bead cultures as previously described by Oppenheimer *et al*. (2014).

### Immunoblot analysis and immunoprecipitation

Protein extracts were maintained in RIPA buffer containing 1 mm phenylmethylsulfonyl fluoride (PMSF; Sigma‐Aldrich), 10 μg mL^−1^ *N*‐Acetyl‐l‐leucyl‐l‐leucyl‐l‐norleucinal, *N*‐Acetyl‐Leu‐Leu‐Nle‐al (ALLN; Sigma‐Aldrich), 1 μm Trichostatin A (TSA), (Sigma‐Aldrich), 5 mm sodium butyrate (Millipore, Billerica, Massachusetts, USA) and protease inhibitor cocktail (Roche, Penzberg, Upper Bavaria, Germany) to inhibit protease activity. For intact and degenerative cartilage, two separate samples were pooled to obtain sufficient protein amounts per immunoblot and immunoprecipitation assay. Protein extracts were run on 10% SDS‐PAGE gel, transferred to a PVDF membrane, and incubated with primary and secondary antibodies in Tris buffered saline (TBS)–Tween 20 solution containing 5% nonfat milk. Nuclear and cytoplasmic protein extracts were isolated using a NE‐PER^™^ kit (Thermo Scientific, Waltham, MA, USA).

Immunoprecipitation with Protein A‐/G‐conjugated agarose beads (Santa Cruz, Dallas, Texas, USA) was carried out in PBS solution supplemented with 1 μm TSA, 10 mm nicotinamide (NAM; Sigma‐Aldrich), and 5 mm sodium butyrate (Millipore) to inhibit protein deacetylase activity. About 1 μm EX‐527 was added to inhibit SIRT1 activity, while 50 μm was added together with 5 mm nicotinamide adenine mononucleotide (NAM) to prevent Sirtuin (SIRT1‐7) activity. About 10 mm nicotinamide adenine dinucleotide (NAD) was added to enhance Sirtuin activity, as indicated.

To examine the role of exportins on SOX9 trafficking, cultured primary human chondrocytes were treated with 10 nm Leptomycin B (Sigma‐Aldrich) for 1 h prior to adding 10 mm NAD. Similarly, 40 μm Importazole (Sigma‐Aldrich) was added 1 h prior to adding the NAD, to analyze the involvement of Importins in SOX9 localization.

Immunoblotting and immunoprecipitation experiments were performed using primary antibodies against β‐actin (Santa Cruz; cat#47778), SIRT1 (Millipore; cat#07‐131), SOX9 (Santa Cruz; cat#20095), Flag (Santa Cruz; cat#807), and Acetyl‐lysine (Abcam, cat#21623, Cambridge, UK), RNA polymerase II (Santa Cruz; cat#9001). Alkaline‐phosphatase (AP)‐conjugated secondary antibodies were used for detection (Anti‐Mouse; Sigma‐Aldrich; cat# A3562; Anti‐rabbit; Sigma‐Aldrich, cat#A3687). Of note, the Acetyl‐lysine antibody (Abcam, cat#21623) is generated against an acetylated KLH conjugate, while putative SOX9 bears KLA and KLW sequences, possibly leading to a weak antigen response of the antibody. To confirm that the acetylated band is indeed SOX9, the immunoprecipitant was run on sodium dodecyl sulfate–polyacrylamide gel electrophoresis (SDS‐PAGE), immunobloted for SOX9, Acetyl‐lysine and aligned with Coomassie gel and processed for in‐gel proteolysis and mass spectrometry analysis (Fig. S1).

Immunoblots were scanned at high resolution, and band intensity was assessed using NIH imagej software (National Institutes of Health, Bethesda, MD, USA). The signal obtained for each protein band was subtracted by the background and normalized to a housekeeping protein (i.e. β‐actin or RNA pol‐II for nuclear extracts).

### In‐gel proteolysis and mass spectrometry analysis

Bands excised from Coomassie blue‐stained SDS‐PAGE gels were sent to the Smoler Proteomics Center at the Technion (Haifa, Israel). The proteins in the gel were reduced with 2.8 mm DTT (60 °C for 30 min), modified with 8.8 mm iodoacetamide in 100 mm ammonium bicarbonate (dark, room temperature for 30 min), and digested in 10% acetonitrile and 10 mm ammonium bicarbonate with modified trypsin (Promega, Fitchburg, Wisconsin, USA) at a 1:10 enzyme‐to‐substrate ratio overnight at 37 °C. The resulting tryptic peptides were resolved by reverse‐phase chromatography on 0.075 × 200‐mm fused silica capillary (J&W) packed with Reprosil reversed‐phase material (Dr. Maisch GmbH, Ammerbuch‐Entringen, Germany). The peptides were then eluted with 30 min gradient (5–28% acetonitrile with 0.1% formic acid in water), followed by 15 min gradient (28–95% acetonitrile with 0.1% formic acid in water) and finally 15 min at 95% acetonitrile with 0.1% formic acid in water at constant flow rates of 0.15 μL min^−1^. Mass spectrometry was performed by a Q‐Exactive plus mass spectrometer (QE; Thermo Scientific) in a positive mode using repetitively full MS scan followed by high energy collision dissociation (HCD) of the 10 most dominant ions selected from the first MS scan. Mass spectrometry data were analyzed using proteome discoverer 1.4 software with Sequest (Thermo Scientific) and Mascot (Matrix Science) algorithms against Human database, and against the sequence of SOX9. Results were filtered with 1% false discovery rate.

### Immunohistochemistry and immunofluorescence

Histology sections were generated from non‐OA and OA articular cartilage following 5 days of tissue fixation in 4% formalin and 3 days of decalcification with 4% formic acid/4% HCl. Similarly, E17 embryo hind paws were fixed in 4% formalin, without decalcification. Samples were dehydrated using a graded series of ethanol washes, embedded in paraffin, and sectioned to 5‐μm slices. Sections were digested with 1 mg mL^−1^ hyaluronidase in PBS at pH 6 (Sigma, cat#H3506) for 1 h at 37 °C and stained with DAB substrate kit (cat#DAB057) after overnight incubation with SOX9 primary antibody. ZytoChem Plus HRP polymer anti‐rabbit (cat#ZUC032) was used as a secondary antibody and staining substrate. Negative controls were incubated with secondary antibody alone and counterstained with hematoxylin.

For immunofluorescence, primary adult chondrocytes were cultured on a glass coverslip within 6‐well plates and treated with NAD or EX527, as indicated. Following treatment, cells were rinsed with PBS, fixed in 4% formaldehyde in PBS for 15 min at room temperature. Thereafter, cells were treated with 0.2% Triton X‐100 in PBS for an additional 15 min at 4 °C. After blocking with 1% Bovine serum albumin (BSA) within PBS for 1 h, cells were incubated for 2 h at RT with primary antibody (1:100 dilution in blocking solution). After three washes with PBS, they were incubated for 30 min with a secondary antibody (1:200 dilution, Alexa‐fluor 568 secondary antibody). Finally, slides were incubated with 1 μg mL^−1^ 4′, 6‐diamidino‐2‐phenylindole (DAPI; Sigma‐Aldrich), washed with PBS, and mounted (Cat# 00‐4958‐02; eBioscience, San Diego, CA, USA) for visualization using an inverted fluorescent microscope (Zeiss, model AXIO, Oberkochen, Germany) with appropriate emission/excitation parameters.

### qRT–PCR

mRNA was isolated using RNeasy mRNA purification columns (Qiagen, Venlo, Netherlands). cDNA was prepared using the OneStep RT–PCR kit (Invitrogen, Waltham, Massachusetts, USA). qPCRs were performed using an ABI qPCR model 7300 or 7900 (Applied Biosystems, Waltham, Massachusetts, USA) with purified samples containing a SYBER Green mix (Applied Biosystems). Primers were as follows: *SIRT1*‐forward: CAGATTAGTAGGCGGCTTGA; reverse: CTAA ACTTGGACTCTGGCAT; *COL2A1* (Exon 2)‐forward: GAGCCCTGCCGGATC TGT; reverse: GAGGCAGTC TTTCACGTCTTC; Matrix Metallopeptidase 13 (*MMP13*)‐forward: AGTTTGCAGAGCGCTACCTGAGAT; reverse: TTTGCCAGT CACCTCTAAGCCGAA; *ADAMTS5*‐forward: TTCAACGTCAAGCCATGGCAA CTG; reverse: TGACGATAGGCAAACTGCACTCCT; *SOX9*‐forward:AGGCAA GCAAAGGAGATGAA; reverse: TGGTGTTCTGAGAGGCACAG; *ACAN*‐forward: TGCGGGTCAACAGTGCCTATC; reverse: CACGATGCCTTTCACCAC GAC. Values were normalized to those obtained for human beta 2‐microglobulin (*B2MR*) or glyceraldehyde 3‐phosphate dehydrogenase (*GAPDH*), which were unaffected by the experimental treatments: *B2MR*‐forward: ACCCCCACTGAAAAAGATG AG; reverse: ATCTTCAAACCTCCATGATGC; *GAPDH*‐forward: TACTAGCGGT TTTACGGGCG; reverse: TCGAACAGGAGCAGAGAGCGA. Mouse *Gapdh:* forward: TGCCCCCATGTTTGTGATG; reverse: TGTGGTCATGAGCCCTTCC. Mouse *Acan*‐forward: GCTGGCTGACCAGACAGTCA; reverse: CCGGATTC CGTAGGTTCTCA.

### Chromatin Immunoprecipitation (ChIP)

Chromatin was cross‐linked using 1% formaldehyde for 10 min. Cells were then lysed (lysis buffer; 10 mm EDTA, 50 mm Tris–HCl, pH 8.0, 1% SDS) and sonicated (Vibra Cell TM; Sonics & Materials, Inc, Newtown, CT, USA) at 35 cycles of 95% amplitude for 30 s, followed by a 45‐s incubation on ice. Samples were then centrifuged, and supernatants were collected. Eluants were treated with RNase A (1 mg mL^−1^, 30 min, 37 °C) and Proteinase K (1 mg mL^−1^ for 1 h at 42 °C) and then DNA was extracted using a Qiaquick spin kit (Qiagen, cat#28104) (Dvir‐Ginzberg *et al*., 2008; Oppenheimer *et al*., 2014). Real‐time PCRs (qPCR) were carried out using primers flanking the −10‐kb *ACAN* enhancer. *ACAN* Enhancer (NM_001844.4)‐forward: ATGTGTCTCAAGTCCA GAATGGAA; reverse: GAAATTCCTTTAGCGGCAACGCCT.

As a positive control, RNA polymerase II (Santa Cruz; cat#9001) was immunoprecipitated and tested for binding to the *GAPDH* promoter using forward (TACTAGCGGTTTTACGGGCG) and reverse (TCGAACAGGAGGAGCAGAGAGCGA) primers. As a negative control, we subjected our samples to the ChIP‐qPCR Human *IGX1A* Negative Control (Qiagen), which targets an ORF‐free intergenic region lacking any known or predicted structural genes.

### Statistical analysis

All experiments were performed on multiple donor samples (*n* ≥ 4 per experiment), as indicated in each figure legend. Statistical analysis was obtained using nonparametric Mann–Whitney analyses. *P*‐values of plotted data equaling 0.05 or less were considered statistically significant.

## Results

### SOX9 protein acetylation levels are higher in degenerative than intact articular cartilage

**Figure 1:**
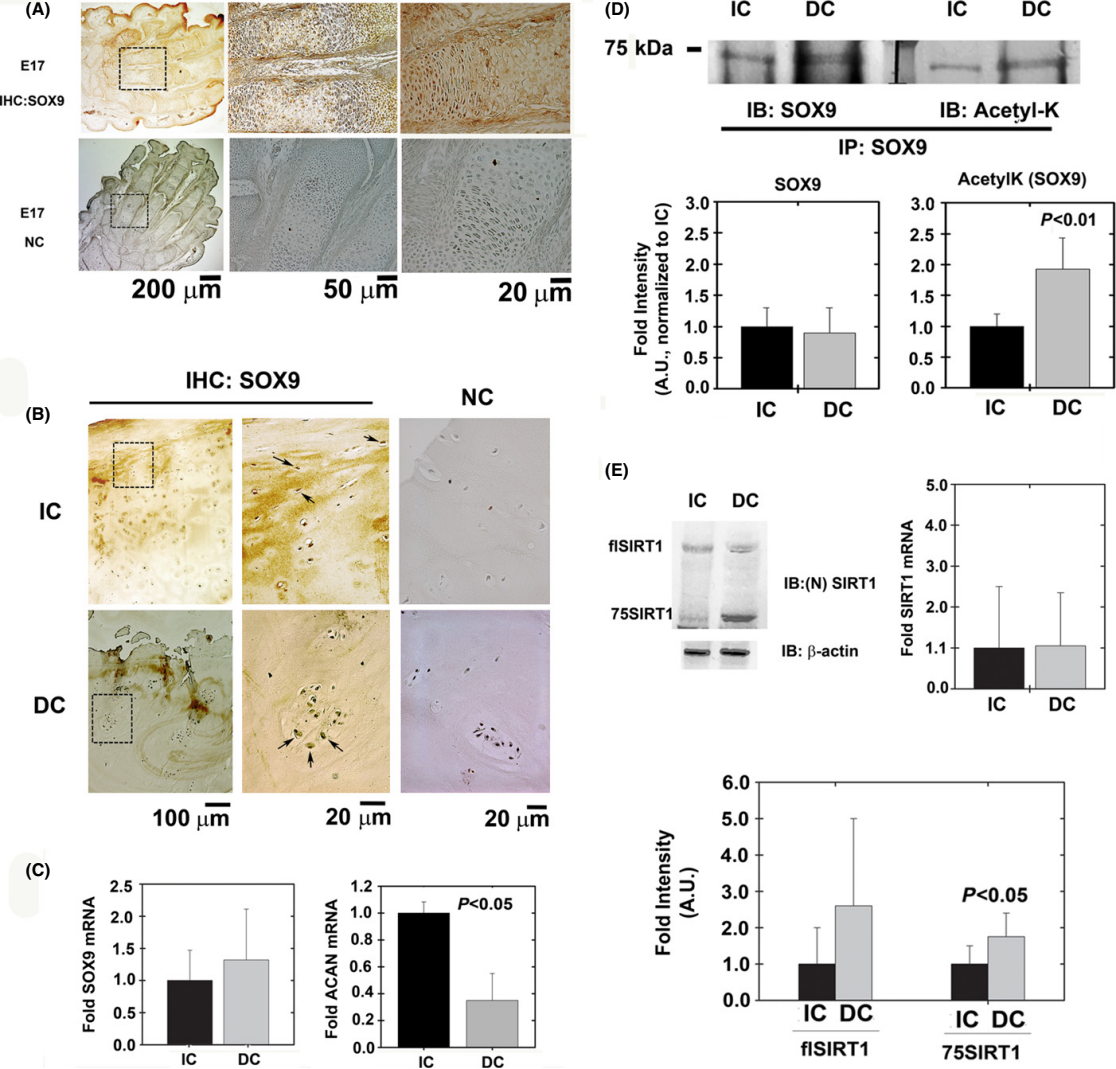
SOX9 levels in healthy and diseased articular cartilage. (A) Sagittal sections of E17 hind paws served as positive and negative controls (NC) for immunohistochemistry (IHC) protocols to detect SOX9 protein levels (*n* = 5).(B) OA‐derived intact cartilage (IC) and degenerated cartilage (DC) were analyzed for SOX9 levels using immunohistochemistry (*n* = 10). (C) *SOX9* and *ACAN* mRNA levels of freshly isolated chondrocytes from IC and DC samples (*n* = 8). As indicated in the Materials and methods section, GAPDH was used as housekeeping gene. (D) SOX9 from IC and DC samples was immunoprecipitated and blotted for SOX9 and acetyl‐lysine (AcetylK; n = 5). (E) Freshly isolated chondrocytes from IC and DC cartilage were analyzed with an *N*‐terminally reactive antibody for SIRT1 to identify its cleavage (*n* = 9). Full‐length SIRT1 is denoted as flSIRT1, while 75SIRT1 is the 75 kDa cleaved variant generated by cathepsin B‐mediated cleavage of SIRT1 on its C‐terminal domain. Right graphs shows mRNA expression for SIRT1 in freshly isolated chondrocytes from IC and DC samples (*n* = 8). Image J was employed to quantify band intensity of all immunoblots. SOX9 acetylation was calculated based on the intensity of acetyl‐lysine (AcetylK) band divided by the band intensity of normalized SOX9, assuming the immunoprecipitate consists mainly of SOX9. Statistical significance was determined based on Mann–Whitney *U*‐test, assuming a *P* < 0.05 to be statistically significant.

We initially sought out to examine SOX9 levels in adult articular cartilage by immunostaining. To this end, we used mouse embryo hind paws, as positive control, to detect high levels of SOX9 in chondrocytes (Fig. 1A). In adult articular cartilage samples, we did not detect significant differences in SOX9 staining between degenerated and intact human articular cartilage (Fig. 1B). However, chondrocyte clusters, which were more abundant in degenerated cartilage, showed diffused staining for SOX9 within the cell (arrows in Fig. 1B). Analysis of intact cartilage (IC) and degenerated cartilage (DC) from OA donors showed that SOX9 mRNA (Fig. 1C, left panel) and protein (Fig. 1D) levels did not significantly differ among these regions, in consistence with histological observations. Although its level appeared unchanged, SOX9 protein was more efficiently acetylated in primary chondrocytes from DC than IC (Fig. 1D). Interestingly, this increase in SOX9 acetylation in DC chondrocytes correlated with lower *ACAN* mRNA levels (Fig. 1C, right panel). We previously showed that SIRT1 activity declines due to cleavage of the protein during OA pathogenesis and cartilage aging (Dvir‐Ginzberg *et al*., 2011; Gabay *et al*., 2012; Oppenheimer *et al*., 2012). The cleavage of SIRT1 was also increased in DC compared with IC (Fig. 1E), whereas SIRT1 mRNA levels were identical among these tissues (Fig. 1E, right panel). This result thus prompted us to test whether SIRT1 is a major contributor of the altered level of SOX9 acetylation in OA cartilage.

### SIRT1 is a major contributor of SOX9 deacetylation

**Figure 2:**
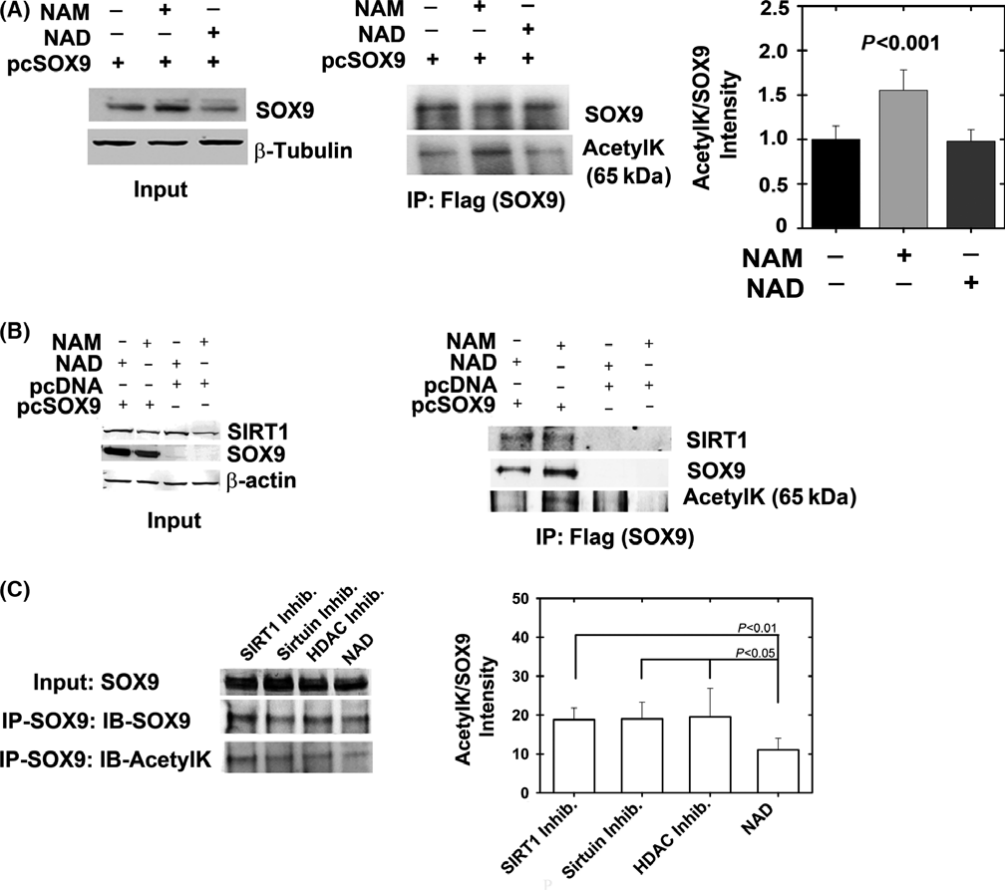
SIRT1 binds and predominantly deacetylates SOX9. HEK293 cells were transfected with a SOX9 expression plasmid (pcSOX9) or pcDNA control and incubated for 24 h with NAD (cofactor of sirtuins) or NAM (a noncompetitive inhibitor of sirtuins). (A) Following immunoblotting for inputs to verify SOX9 expression (right panel), the extracts were immunoprecipitated for flag‐tag and immunoblotted for SOX9 and acetylated lysine (AcetylK), (*n* = 5). Semi‐quantitative band intensity for acetylated SOX9 is presented in the right graph in A. (B). Left panel shows Flag (SOX9) immunoprecipitants possessing augmented binding to endogenous SIRT1 only when SOX9 was overexpressed. Further, SOX9 protein was not acetylated upon treatment with NAD (sirtuin cofactor) compared with NAM (sirtuin inhibitor) treatment (*n* = 5). (C) HEK293 cells were transfected with SOX9 expression plasmid and incubated with 1 μm EX‐527 (SIRT1 inhibitor), 10 mm NAM and 50 μm EX‐527 (Sirtuin inhibitors), 10 mm NAM/50 μm EX‐527/1 μm TSA/5 μm Sodium butyrate (HDAC inhibitors), and 10 mm NAD (Sirtuin cofactor). SOX9 was immunoprecipitated and blotted for SOX9 and acetyl‐lysine (*n* = 7). ImageJ was used for quantification of band intensity (right panel of B) and shows no change in SOX9 acetylation among the inhibitor treatments, confirming that SIRT1 is predominantly responsible for SOX9 deacetylation.

To establish SOX9 basal acetylation level, SOX9 was expressed (pcSOX9) in HEK293 cells which were either untreated or treated with NAD or NAM (Fig. 2A). It should be noted that while HEK293 cells express SIRT1 endogenously, they express very little SOX9, which is the reason for transfecting SOX9 ectopically. The data show that SOX9 acetylation is similar between untreated and NAD‐treated cells, but are enhanced 50% upon NAM treatment (Right graph of Fig. 2A). Next, we aimed to assess whether SOX9 deacetylation is achieved by SIRT1 or other HDACs or sirtuins. We transfected HEK293 cells with SOX9, immunoprecipitated SOX9, and assessed its acetylation state using an acetyl‐lysine (AcetylK) antibody, similar to Fig. 2A. The total level of SOX9 protein was increased upon transfection, as shown in input material immunoblots, but acetylation of the protein was detected only in cells treated with NAD (sirtuin cofactor) but not in cells treated with NAM (sirtuin inhibitor) (Fig. 2B). Interestingly, SIRT1 co‐immunoprecipitated with SOX9 in extracts from both types of cells (Fig. 2B). Together, these findings suggested that SIRT1 forms a complex with SOX9 independently of NAM/NAD treatment, but deacetylates SOX9 only in the presence of its cofactor. To confirm that SOX9 is indeed acetylated, we overexpressed it in HEK293 cells, treated the cells with for NAD or the SIRT1 inhibitor EX527, and carried out LC‐MS analysis to identify SOX9 (Fig. S1 ). Data show unique SOX9 peptides identified in all eight runs.

Given that NAM and NAD are capable of affecting the activity of multiple deacetylases in addition to SIRT1, we examined whether other sirtuins or HDACs could potentially deacetylate SOX9. To this end, we incubated SOX9‐overexpressing HEK293 cells with EX527 (1 μm) to specifically inhibit SIRT1; with 10 mm NAM/50 μm EX‐527 to inhibit all sirtuins; and with 10 mm NAM/50 μm EX‐527/1 μm TSA/5 μm sodium butyrate to inhibit all HDACs and sirtuins (Fig. 2C). As a control, we incubated cells with 10 mm NAD to enhance sirtuin activity. Results showed no significant changes in SOX9 acetylation upon inhibiting all sirtuins or HDACs compared with selectively inhibiting SIRT1. Thus, the data suggest that SIRT1 may be predominantly responsible for SOX9 deacetylation.

### 3D‐cultured chondrocytes display SOX9 hypo‐acetylation and augmented ACAN mRNA levels compared with monolayer cultures

**Figure 3:**
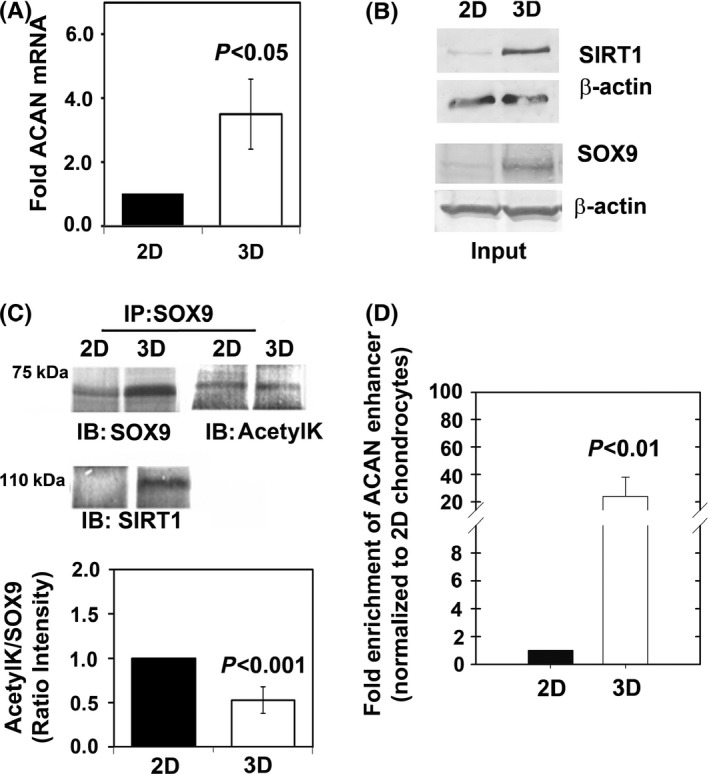
3D‐cultured chondrocytes present hypo‐acetylated SOX9 and higher ACAN expression levels. OA‐derived articular chondrocytes were cultured in monolayer (2D) or encapsulated in alginate (3D), (*n* = 5). (A) *ACAN* RNA expression levels are higher in 3D‐cultured chondrocytes (*n* = 5). (B) Protein levels of SOX9 and SIRT1 are augmented in 3D cultures (*n* = 5). (C) SOX9 was immunoprecipitated and immunobloted for SOX9, SIRT1, and acetyl‐lysine (AcetylK), (*n* = 5). Results show SOX9‐SIRT1 complex is formed in 3D cultures and that SOX9 is hypo‐acetylated in 3D culture settings. (D) ChIP analysis for −10 kb upstream *ACAN* enhancer was carried out for 2D and 3D cultures (*n* = 5), after normalizing the values to input and validating negligibility in negative controls (see Materials and methods).

Given the spherical morphology of chondrocytes in native tissue and propensity of chondrocytes to dedifferentiate in monolayer culture, various biomimetic 3D‐matrix techniques have been used to maintain the chondrocyte natural morphology. As shown previously, osteoarthritic cartilage‐derived primary chondrocytes cultured in alginate beads were expressing cartilage‐specific genes, including ACAN, at high level (Liu *et al*., 1998; Oppenheimer *et al*., 2014) (Fig. 3A). Compared with monolayer cultures, 3D cultures displayed high levels of SIRT1 and SOX9 protein (Fig. 3B), and a low level of SOX9 acetylation (Fig. 3C). Moreover, a ChIP assay (−10 kb *ACAN* enhancer) demonstrated a much higher efficiency of SOX9 binding to a −10‐kb cartilage‐specific *ACAN* enhancer in cells cultured in alginate beads than in cells grown in monolayer (Fig. 3D). These data suggest for the first time that reduced acetylation might promote SOX9 binding to one of its genomic targets and thereby enhance gene transactivation.

### Mechanical loading does not enrich SOX9 binding to ACAN enhancer

Mechanical loading has been shown to effect proteoglycan metabolism in cartilage explants (Sauderland *et al*., 2003). To understand whether mechanical stimulus impacts SOX9 acetylation and *ACAN* transactivation, we cultured chondrocytes in 3D alginate beads and applied hydrostatic load by centrifugation (0.05 MPa 30 min^−1^, 0.1 MPa 30 min^−1^, 0.1 MPa 5 min^−1^). The physiologically relevant compressive hydrostatic pressure of articular cartilage upon walking activity is approx. 1 MPa or less (Detzel & Van Wie, 2011; Jeon *et al*., 2012; Hilz *et al*., 2014). To this end, we designed an experimental setting applying intermittent low hydrostatic loads (i.e. 0.05 or 0.1 MPa). Applying higher loads disrupted the structure of the 3D alginate microbeads and caused cells to be released in the course of loading (data not shown).

**Figure 4:**
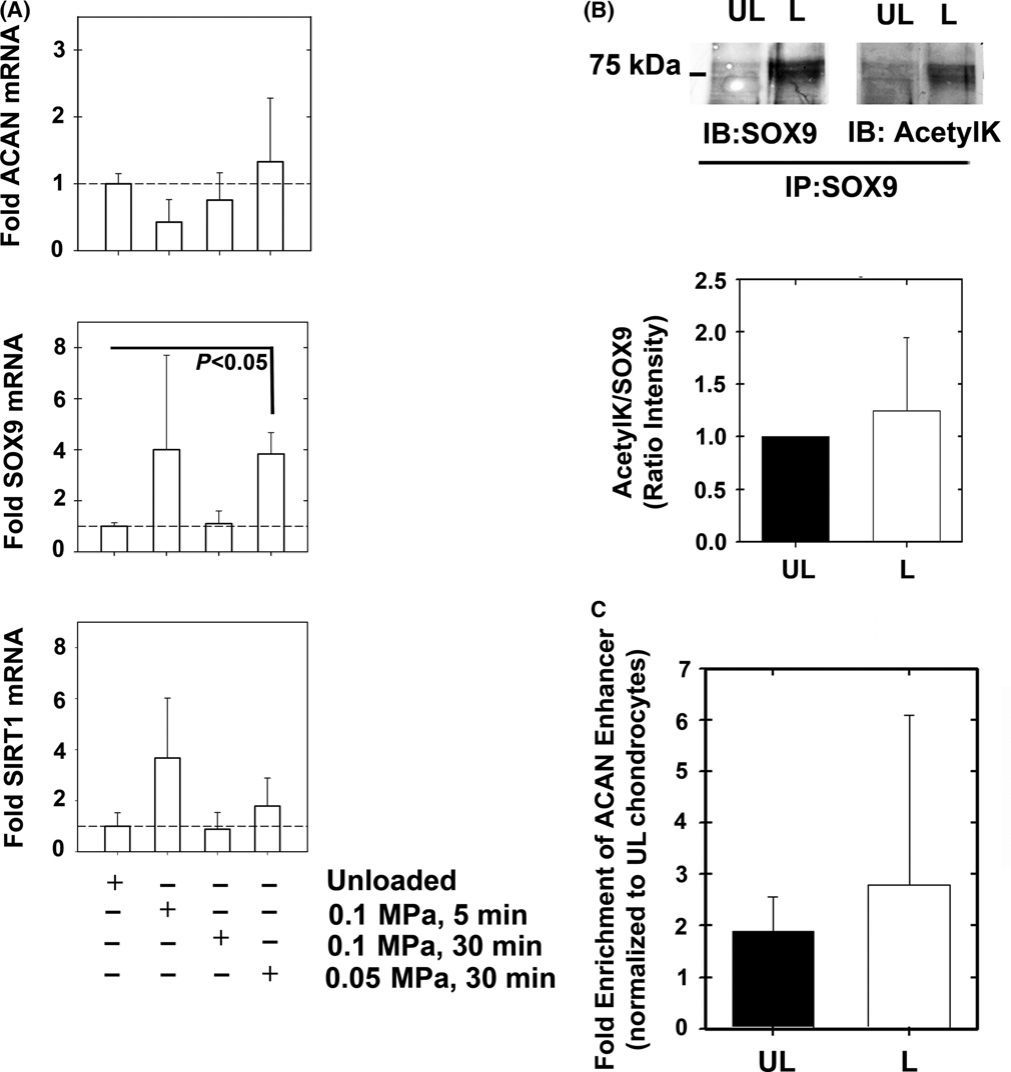
Gene expression of alginate encapsulated chondrocytes under hydrostatic load conditions. Chondrocytes were encapsulated in alginate beads and hydrostatically loaded via centrifugation (0.05 MPa 30 min^−1^, 0.1 MPa 30 min^−1^, 0.1 MPa 5 min^−1^). Gene expression of (A) *ACAN, SOX9*, and *SIRT1* were assessed (*n* = 5). (B) Immunoprecipitating SOX9 from 0.05MPa 30 min^−1^ loading conditions (L) vs. unloaded (UL) conditions showed increased SOX9 expression upon loading (*n* = 6). The SOX9 immunoprecipitants were immunoblotted for SOX9 and acetyl‐lysine (AcetylK) and quantified for the extent of SOX9 acetylation (lower panel of B, *n* = 6). (C) ChIP analysis from UL and L samples did not show changes in SOX9 binding among the treatments (*n* = 7).

*SOX9* expression was found to be increased during long hydrostatic load protocols (0.05 MPa, 30 min), but *ACAN, COL2A1, ADAMTS5, MMP13* and *SIRT1* expression was not (Fig. 4A, Fig. S2). In line with the unaffected *COL2A1* levels that we report herein, Wolf *et al*. reported that the rate of *COL2A1* synthesis is unaffected by intermitted mechanical loading in cartilage explants (Wolf *et al*., 2007). As mentioned, *SOX9* expression was higher following loading at 0.05 MPa for 30 min than under unloaded conditions (fourfold; Fig. 4A). SOX9 protein level was increased upon loading, a result consistent with mRNA findings, but SOX9 acetylation level was unchanged (Fig. 4B, lower panel). Moreover, ChIP data assay showed no significant change in SOX9 binding to the −10 kb ACAN enhancer, a result consistent with the unchanged *ACAN* expression level (Fig. 4C).

In summary, albeit application of mechanical loading increased the mRNA and protein levels of SOX9, we did not observe increased levels of expression of its *ACAN* target nor did we observe significant changes in SOX9 binding to the −10 kb ACAN enhancer. The ratio of acetyl‐SOX9 to SOX9 protein level was unchanged between loaded and unloaded samples, possibly explaining why SOX9 binding to the ACAN enhancer was unaffected too. Hence, these results further suggest that reduced SOX9 acetylation is required for SOX9 binding to the *ACAN* enhancer.

### SOX9 acetylation state does not affect protein stability but prevents SOX9 nuclear entry

**Figure 5:**
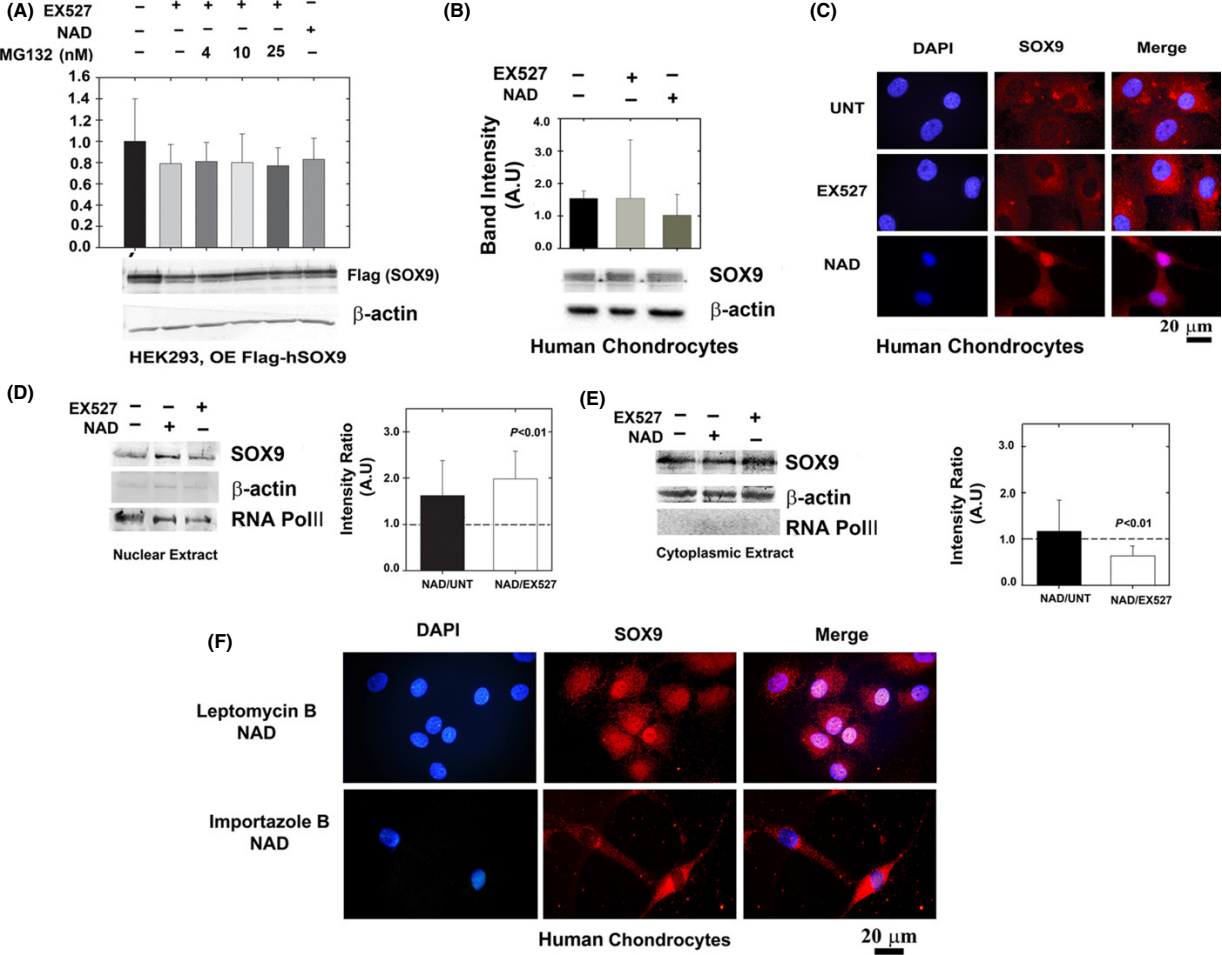
Deacetylated SOX9 does not exhibit enhanced protein stability but possesses enhanced nuclear localization. (A) HEK293 cells were transfected with Flag‐SOX9 expression plasmid (OE; overexpression) and treated with NAD, EX527 and MG132. Levels of SOX9 were monitored by immunoblotting of flag‐tag (*n* = 5). (B) Cultured human chondrocytes were immunoblotted for SOX9 levels in the presence of EX527 or NAD (*n* = 5). (C) Confocal microscopy of primary cultured chondrocytes treated as in B (*n* = 5). Blue florescence for nuclear staining via DAPI; red florescence for staining of endogenously expressed SOX9 via anti‐SOX9 antibody and Alexa‐fluor 568 secondary antibody. Purple florescence indicates overlap of blue and red and implies enhanced SOX9 levels in the nuclear compartment upon NAD treatment. ‘UNT’ denotes untreated cells. The images were magnified x100. (D) Nuclear and (E) cytoplasmic extracts of SOX9 in EX527, NAD and untreated human cultured chondrocytes (*n* = 5). Semi‐quantitative analysis of RNA POL‐II and β‐actin from nuclear extracts indicates a 9.9% contamination of cytoplasmic proteins, while cytoplasmic extracts possessed a 13% contamination of nuclear proteins, overall indicating that the level of enrichment is significantly high in these extracts. (F) Human chondrocytes cultured on coverslip and treated with 10 nM Leptomycin B and 10 mm NAD or 40 μm Importazole and NAD. Immunostaining and capture of images as indicated in C.

To explain why SOX9 binds *ACAN* enhancer upon deacetylation, we examined whether SOX9 stability or trafficking could be affected due to this protein modification (Fig. 5). Given that SOX9 transcription activity was reduced when hyperacetylated (Fig. 3), we postulated that a hyperacetylated state may interfere with SOX9 protein stability. To modulate SOX9 acetylation, we used SIRT1 inhibitor EX527, rendering it hyperacetylated state, while use of NAD, a sirtuin cofactor, rendered SOX9 hypo‐acetylated. Protein stability was examined in HEK293 cells overexpressing SOX9 subjected to EX527 and NAD treatment, in the presence of MG132 (proteasome inhibitor; Fig. 5A), assuming that adding MG132 would compensate for reduced protein stability during SOX9 hyperacetylation, rendered by EX527. In general, there were no significant changes in SOX9 protein levels in HEK293 cells or cultured human chondrocytes, upon NAD or EX527 treatment (Fig. 5A,B, respectively). Administering EX527 and MG132 did not alter SOX9 protein levels, even at higher doses of MG132. Therefore, it appears that SOX9 stability is not influenced by its acetylation state.

Next, we examined nuclear localization of SOX9 in cultured primary human chondrocytes displayed a significant increase in nuclear localization of SOX9 in NAD‐treated compared with EX527‐treated cells and untreated controls as judged by confocal visualization of stained cells (Fig. 5C). These data were in line with cytoplasmic and nuclear extracts showing enhanced SOX9 in the nucleus with less cytoplasmic SOX9 in NAD‐treated cultured chondrocytes, when comparing with EX527‐treated chondrocytes (Fig. 5D,E). Overall, it appears that NAD enhances nuclear localization of SOX9, possibly through enhanced SIRT1 deacetylation.

**Figure 6:**
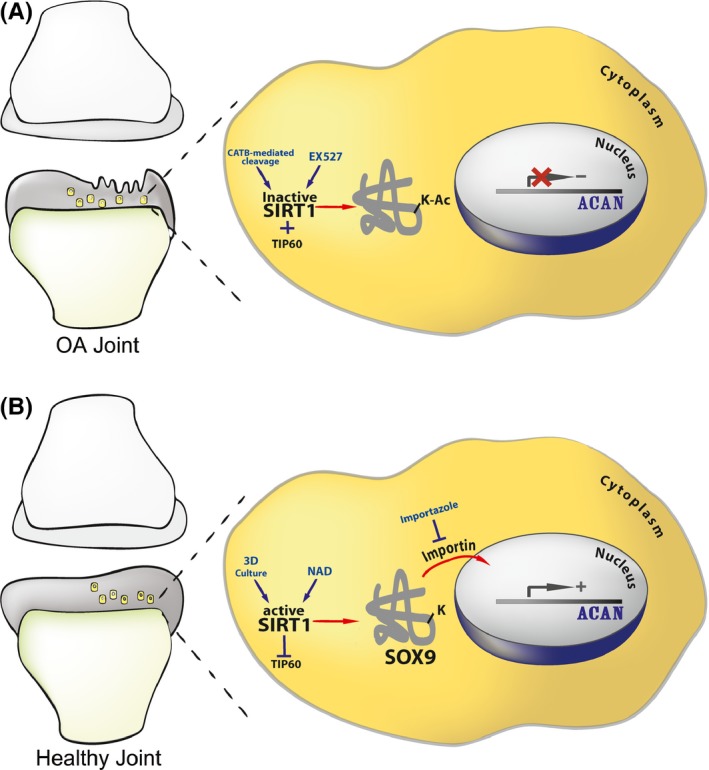
Scheme illustrating the mechanism of SOX9 nuclear entry. The illustration shows how acetylation state could affect SOX9 nuclear entry and ACAN expression in intact and OA cartilage. (A) Proposed mechanism in a degenerating OA joint, wherein cells are less capable of expressing ACAN. This is due to an increase in the acetylation state of SOX9 succeeding SIRT1 inactivation. SIRT1 inactivation is exerted by cathepsin B (CATB)‐mediated cleavage (Dvir‐Ginzberg *et al*., 2011) or incubation with the SIRT1 inhibitor EX527. In addition to SIRT1 inactivation, TIP60 acetyl‐transferase activity will contribute to SOX9 hyperacetylation (Hattori *et al*., 2008). (B) Healthy articular cartilage wherein SIRT1 is active. SIRT1 activity is rendered by 3D culture conditions and the presence of SIRT1 activators (i.e. resveratrol, NAD, etc). Enhanced SIRT1 activity will inhibit TIP60 (Wang & Chen, 2010), thereby promoting SOX9 hypo‐acetylated state. Hypo‐acetylated SOX9 is capable of entering the nuclear compartment and binding the enhancer of ACAN to induce gene transactivation and expression. The import of hypo‐acetylated SOX9 to the nuclear compartment is facilitated by importin β, which is specifically inhibited by importazole.

To further assess whether changes in SOX9 nuclear transport proteins are effected by its acetylation state, we used inhibitors of nuclear transport proteins. To this end, we used leptomycin B which inhibits CRM1/exportin 1 activity and prevents export of nuclear cargo proteins to the cytoplasm, while importazole was used to inhibit importin β activity and prevent import of cargo protein to the nuclear compartment from the cytoplasm. Cultured chondrocytes treated with NAD showed more nuclear localization when treated with leptomycin B as compared to importazole treated cells (Fig. 6F). The data indicate that a deacetylated state of SOX9 may enhance its import, possibly via increased affinity to importin‐β transport receptors, but not due to enhanced export of the deacetylated SOX9 to the cytoplasm by exportin proteins. Taken together, our data show that deacetylation of SOX9 does not affect its stability, rather enhances its trafficking into the nucleus, rendering its access to endogenous ACAN enhancer and possibly other genomic targets.

## Discussion

SOX9 has been identified as a pivotal regulator of chondrogenesis, cartilage growth and maintenance. However, very little is known regarding its roles in adult and aging cartilage. Like many other regulators, we speculated SOX9 is prone to posttranslational modifications which may impact its activity during adulthood and thereby possibly leading to its limited transcriptional capacity. For example, SOX9 was shown to be phosphorylated by PKA on serine 211 rendering its augmented DNA binding to the 18‐bp COL2A1 enhancer sequence (Huang *et al*., 2000). TIP60 was first discovered to target SOX9 for acetylation by Hattori *et al*. (2008) while in parallel SIRT1 was found to deacetylate SOX9 (Dvir‐Ginzberg *et al*., 2008). Interestingly, both studies suggested that acetylation or deacetylation of SOX9 might contribute to cartilage‐specific gene expression. To understand whether SOX9 acetylation correlates with cartilage gene expression and OA, we examined its capacity to bind *ACAN* enhancer region using various experimental settings involving human cultured primary chondrocytes. Here, we find that deacetylation by SIRT1 renders SOX9 enhanced binding to its −10 kb target of *ACAN* leading to its gene expression. Even when SOX9 protein levels were increased upon hydrostatic loading, the unchanged level of SOX9 acetylation, did not alter *ACAN* chromatin binding or render significant changes in *ACAN* expression. Intriguingly, we report for the first time that the change in the ability of SOX9 to bind to the ACAN enhancer site is influenced by SOX9 acetylation state and is facilitated via enhanced nuclear entry following SOX9 deacetylation.

Our results suggest that SIRT1 might be the primary deacetylase rendering SOX9 in a hypo‐acetylated state, similar to its action on various other regulators as β‐catenin, MyoD, and RelA (Fulco *et al*., 2003; Yeung *et al*., 2004; Simic *et al*., 2013). A previous study, by Baltus *et al*. (2009) established that murine SOX2 is acetylated by P300/CBP on lysine 75, and enhanced acetylation renders its proteasomal degradation and impairs its capacity to bind its nuclear target. Moreover, this study shows that the K75 site was located in the nuclear export sequence of SOX2 and was speculated to prevent SOX2 from entering the nucleus, further contributing to its instability (Baltus *et al*., 2009). SRY, another SOX family member, was found to possess enhanced nuclear localization via interacting with importin β upon acetylation (Thevenet *et al*., 2004). Our data do not support that SOX9 acetylation state affects its protein stability; however, we have seen significant nuclear localization upon SOX9 deacetylation in adult human chondrocytes. More relevant to OA, degenerated cartilage displaying higher acetylation of and presented a more diffused staining compared to intact cartilage. The changes in SOX9 acetylation between intact and degenerated cartilage correlate with altered *ACAN* expression, in line with our experimental data. Based on the observations so far, we propose that a deacetylated state of SOX9, facilitated by enhanced SIRT1 activity, enables its import to the nucleus and supports its transcriptional activity and transactivation of ACAN (see illustration Fig. 6).

Very little is known about the functional changes in SOX9 activity, as osteoarthritis develops. Most of the work regarding SOX9 establishes its role in skeletal development until postnatal stages (Bi *et al*., 2001; Dy *et al*., 2012). However, several studies in rat OA models and human healthy and OA samples conclude that SOX9 mRNA levels are extremely low during OA pathogenesis (Haag *et al*., 2008; Kim & Im, 2011; Kim *et al*., 2013), which coincides with our observations in Fig. S3 showing reduced SOX9 protein in OA vs. non‐OA primary human chondrocytes. As we observed in degenerating cartilage, SOX9 was shown in low protein levels but hyperacetylated in actively degenerating regions, indicating it is less likely to bind chromatin and transactivate its targets. Additional data with human samples by Kim *et al*., (2013) establish that SOX9 promoter becomes hypermethylated in the course of OA pathogenesis, contributing to its reduced expression. Moreover, assessing histone modifications upon SOX9 gene showed enhanced levels of repressive histone marks (e.g. H3K9me3 and H3K27me3) and decreased levels of the inductive histone mark H3K9ac in the OA vs. non‐OA samples, leading to repressed SOX9 expression. It appears that SOX9 reduced expression is age dependent and results in gradual loss of the protein.

Our previous reports provide another facet of regulation for SOX9, dependent on the decreased activity of SIRT1 seen with OA development (Dvir‐Ginzberg *et al*., 2011; Gabay *et al*., 2012; Oppenheimer *et al*., 2012; Dvir‐Ginzberg & Steinmeyer, 2013). With advanced age and Inflammtory insult to the joint, SIRT1, the primary deacetylase of SOX9, becomes cleaved and inactivated, potentially leading to enhanced SOX9 acetylation. This mechanism provides another mode of regulation for SOX9 cellular activity, even in its scarcity during OA and with advanced aging. Overall, our investigations show for the first time that SOX9 acetylation prevents its nuclear localization and thereby reduces SOX9 access to *ACAN* and possibly other potential genomic targets.

## Conflict of interest

None declared.

## Supporting information


**Fig. S1** SOX9 identification after treatment with EX527.

**Fig. S2** Gene expression of alginate encapsulated chondrocytes under hydrostatic load conditions.

**Fig. S3** Reduced SOX9 protein in primary human chondrocytes from OA patients.
