# Corrigendum

**DOI:** 10.1111/acel.12609

**Published:** 2017-07-12

Bar Oz, M., Kumar, A., Elayyan, J., Reich, E., Binyamin, M., Kandel, L., Liebergall, M., Steinmeyer, J., Lefebvre, V. and Dvir‐Ginzberg, M. (2016), Acetylation reduces SOX9 nuclear entry *and ACAN* gene transactivation in human chondrocytes. Aging Cell, 15: 499–508. https://doi.org/10.1111/acel.12456

In the article ‘Acetylation reduces SOX9 nuclear entry *and ACAN* gene transactivation in human chondrocytes’, the author would like to correct one sentence in the Results section under the ‘SIRT1 is a major contributor of SOX9 deacetylation’.

The sentence is:

The total level of SOX9 protein was increased upon transfection, as shown in input material immunoblots, but acetylation of the protein was detected only in cells treated with NAD (sirtuin cofactor) but not in cells treated with NAM (sirtuin inhibitor) (Fig. 2B).

It should read as:

The total level of SOX9 protein was increased upon transfection as shown in input immunoblots, but acetylation of the protein was detected only in cells treated with NAM (sirtuin inhibitor) but not in cells treated with NAD (sirtuin cofactor) (Fig. 2B).

The authors would like to apologize for any inconvenience caused.

