# Supplementary figures and images for: Acetylation reduces SOX9 nuclear entry and ACAN gene transactivation in human chondrocytes
Source: Aging Cell. 2016 Feb 22;15(3):499–508. doi: 10.1111/acel.12456 (PMC4854920; doi:10.1111/acel.12456)

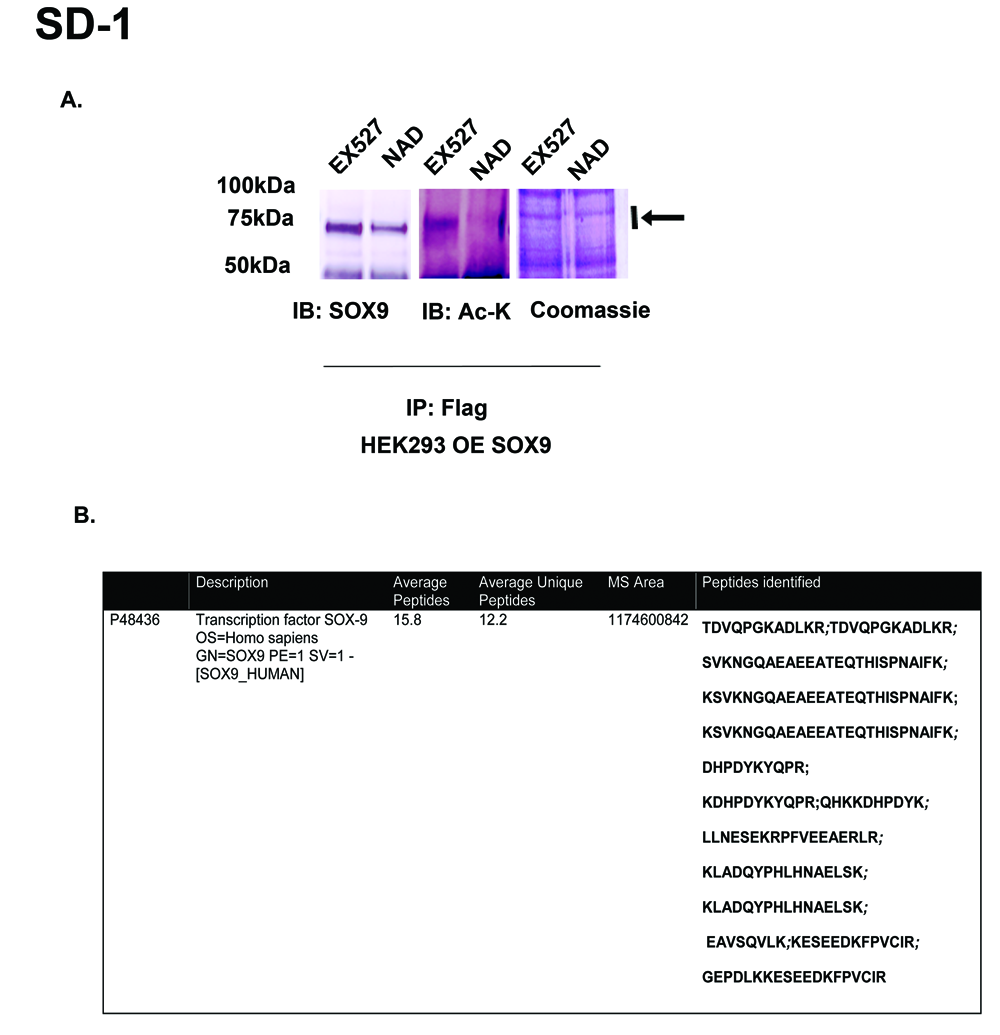

Supplement: Supplementary file 1 — Fig. S1 SOX9 identification after treatment with EX527. [file ACEL-15-499-s001.tif]

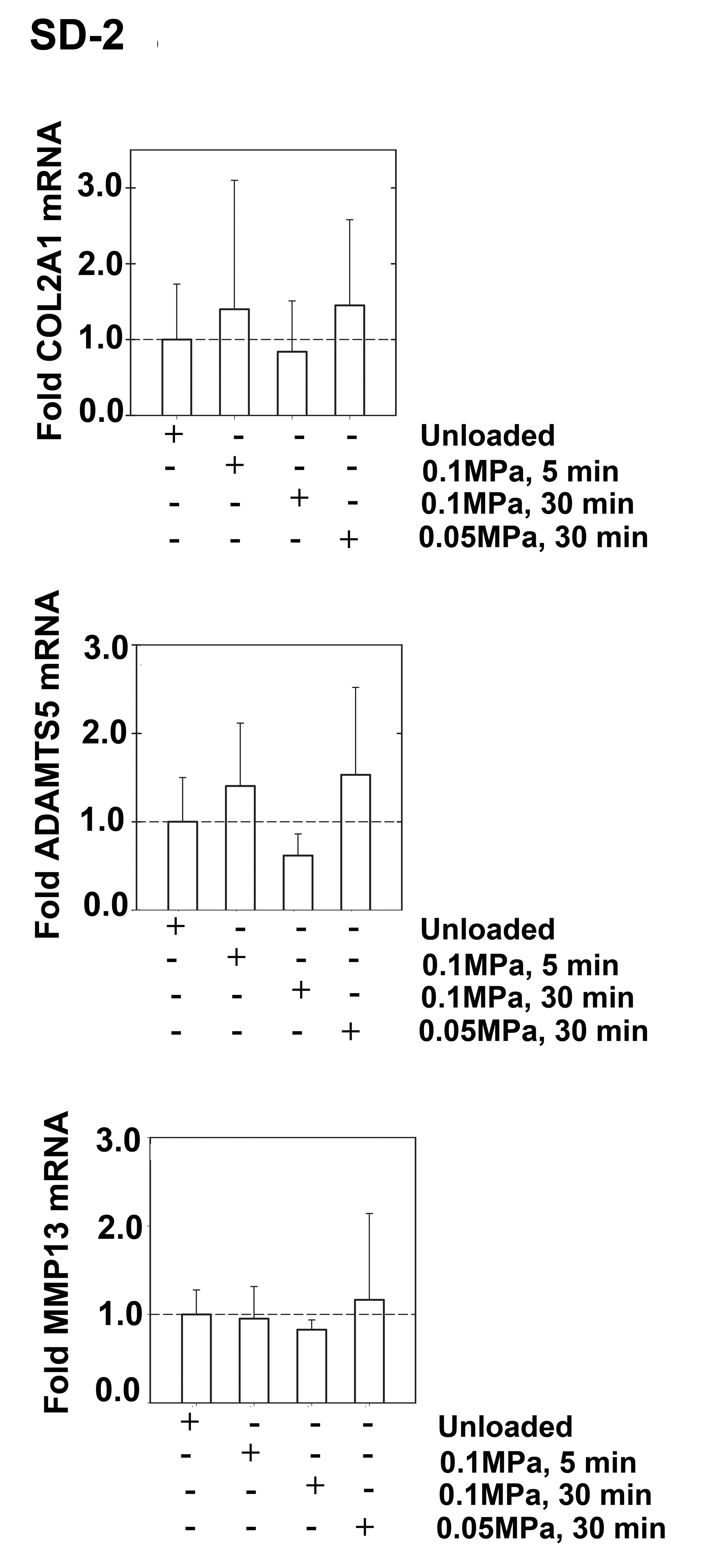

Supplement: Supplementary file 2 — Fig. S2 Gene expression of alginate encapsulated chondrocytes under hydrostatic load conditions. [file ACEL-15-499-s002.tif]

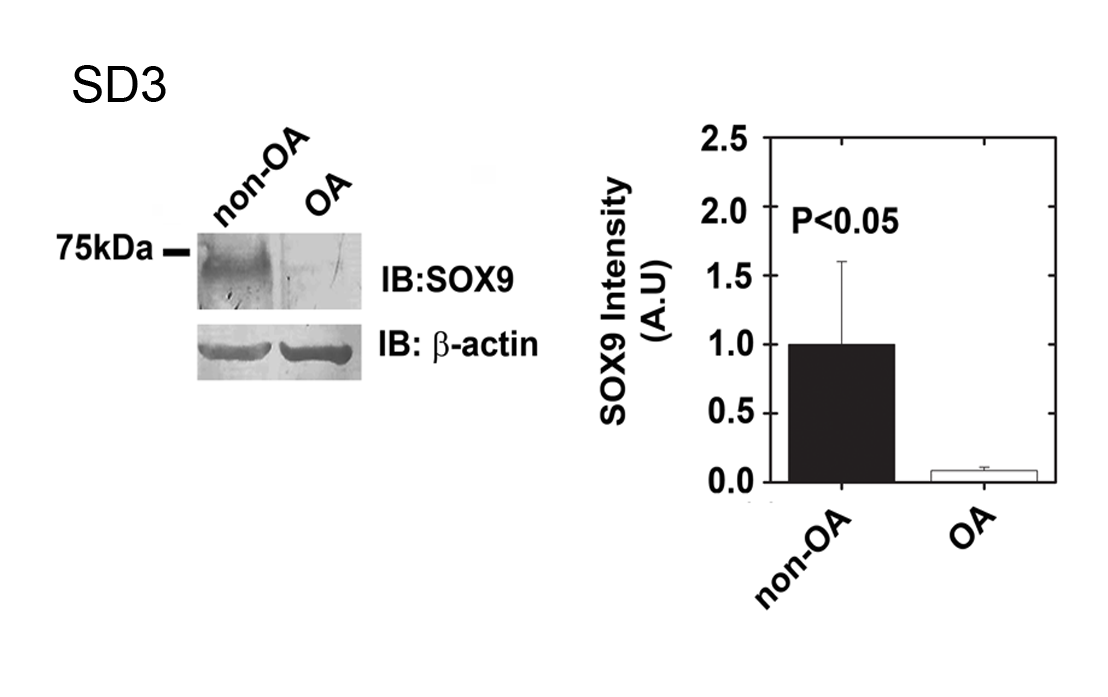

Supplement: Supplementary file 3 — Fig. S3 Reduced SOX9 protein in primary human chondrocytes from OA patients. [file ACEL-15-499-s003.tif]
